# Effectiveness of Cloth Masks for Protection Against Severe Acute Respiratory Syndrome Coronavirus 2

**DOI:** 10.3201/eid2610.200948

**Published:** 2020-10

**Authors:** Abrar A. Chughtai, Holly Seale, C. Raina Macintyre

**Affiliations:** University of New South Wales, Kensington, New South Wales, Australia (A. Chughtai, H. Seale, C.R. Macintyre);; Arizona State University, Tempe, Arizona, USA (C.R. Macintyre)

**Keywords:** 2019 novel coronavirus disease, coronavirus disease, COVID-19, severe acute respiratory syndrome coronavirus 2, SARS-CoV-2, viruses, respiratory infections, zoonoses, face masks, masks, respirators, cloth masks

## Abstract

Cloth masks have been used in healthcare and community settings to protect the wearer from respiratory infections. The use of cloth masks during the coronavirus disease (COVID-19) pandemic is under debate. The filtration effectiveness of cloth masks is generally lower than that of medical masks and respirators; however, cloth masks may provide some protection if well designed and used correctly. Multilayer cloth masks, designed to fit around the face and made of water-resistant fabric with a high number of threads and finer weave, may provide reasonable protection. Until a cloth mask design is proven to be equally effective as a medical or N95 mask, wearing cloth masks should not be mandated for healthcare workers. In community settings, however, cloth masks may be used to prevent community spread of infections by sick or asymptomatically infected persons, and the public should be educated about their correct use.

 As a result of the coronavirus disease (COVID-19) pandemic, supplies of medical masks and respirators are limited globally. Medical/surgical masks and respirators are commonly used as protection against respiratory and other infections. The main difference in these 2 products is the intended use. Medical masks are used in both healthcare and community settings to protect from droplet infections and from splashes and sprays of blood and body fluids. They are also used to prevent the spread of infection from sick or asymptomatic persons (also referred to as source control). Respirators are fit around the face, designed for respiratory protection, and used mostly in healthcare settings. 

Heated debate surrounds healthcare workers having to either reuse or extend the use of disposable products, sterilize their respirator, or resort to wearing cloth or other homemade masks (*1*,*2*). Historically, cloth masks have been used to protect healthcare workers and the general public from various respiratory infections (*3*). However, most studies of cloth masks were conducted in vivo and during the first half of the 20th century, before medical masks were developed. To our knowledge, only 1 randomized controlled trial has been conducted to determine the efficacy of cloth masks (*4*). In this article, we discuss the evidence to inform the use of cloth masks for prevention of respiratory infections and propose strategies for cleaning and decontamination to protect frontline healthcare workers and the general public.

## Historical Use of Cloth Masks

During the early 20th century, various types of cloth masks (made of cotton, gauze, and other fabrics) were used in US hospitals. Rates of respiratory infections among healthcare workers who used masks made of 2–3 layers of gauze were low (*5*). Cloth masks were also used to protect healthcare workers from diphtheria and scarlet fever. During the 1918 Spanish influenza pandemic, masks made of various layers of cotton were widely used by healthcare workers and the general public. Gauze masks were used during the second Manchurian plague epidemic in 1920–1921 and a plague epidemic in Los Angeles in 1924; infection rates among healthcare workers who wore masks were low (*6*). During the 1930s and 1940s, gauze and cloth masks were also used by healthcare workers to protect themselves from tuberculosis (*7*). In the middle of the 20th century, after disposable medical masks had been developed, use of cloth masks decreased; however, cloth mask use is still widespread in many countries in Asia. During the outbreak of severe acute respiratory syndrome in China, cotton masks were widely used by healthcare workers and the general public, and observational studies found them to be effective (*8*).

## Studies of Cloth Mask Efficacy

In 2015, we conducted a randomized controlled trial to compare the efficacy of cloth masks with that of medical masks and controls (standard practice) among healthcare workers in Vietnam (*4*). Rates of infection were consistently higher among those in the cloth mask group than in the medical mask and control groups. This finding suggests that risk for infection was higher for those wearing cloth masks. The mask tested was a locally manufactured, double-layered cotton mask. Participants were given 5 cloth masks for a 4-week study period and were asked to wash the masks daily with soap and water (*4*). The poor performance may have been because the masks were not washed frequently enough or because they became moist and contaminated. Medical and cloth masks were used by some participants in the control group, but the poor performance of cloth masks persisted in post hoc analysis when we compared all participants who used medical masks (from the control and the medical mask groups) with all participants who used only a cloth mask (from the control and the cloth mask groups)(*4*).

We also examined the filtration ability of cloth masks by reviewing 19 studies (*3*). We found that the filtration effectiveness of cloth masks is generally lower than that of medical masks and respirators. Filtration effectiveness of cloth masks varies widely; some materials filter better than others (*9*–*11*). Filtration effectiveness of cloth masks depends on many factors, such as thread count, number of layers, type of fabric, and water resistance (*3*). One study tested medical masks and several household materials for the ability to block bacterial and viral aerosols. Participants made masks from different materials, and all masks tested showed some ability to block the microbial aerosol challenges although less than that of medical masks (*11*). Another study found that homemade cloth masks may also reduce aerosol exposure although less so than medical masks and respirators (*12*). Masks made of cotton and towel provide better protection than masks made of gauze. Although cloth masks are often not designed to fit around the face, some materials may fit snugly against the face. One study found that the use of nylon stockings around the mask improved filtration (A.V. Mueller et al., unpub. data, https://www.medrxiv.org/content/10.1101/2020.04.17.20069567v2.full.pdf). Filtration effectiveness of wet masks is reportedly lower than that of dry masks (*3*).

## Policies and Guidelines Associated with Cloth Mask Use

Despite common use of cloth masks in many countries in Asia, existing infection control guidelines do not mention their use (*13*). However, some previous infection control guidelines have discussed use of cloth masks when medical masks and respirators are not available. For example, in an infection control guideline developed in 1998, the US Centers for Disease Control and Prevention (CDC) and the World Health Organization (WHO) recommended using cotton masks to protect from viral hemorrhagic fevers in low-resource healthcare settings in Africa if respirators or medical masks were not available (*14*). Similarly, WHO also discussed the option of using cloth masks to protect wearers from acquiring infection during the 2009 influenza A(H1N1) pandemic (*15*). In 2006, the US Institute of Medicine, National Academy of Sciences, prepared a report about the reusability of face masks during an influenza pandemic (*16*). The members were hesitant to advise against the use of cloth masks because of high mask demand during pandemics (*16*). As a result of the shortage of masks during the recent COVID-19 pandemic, CDC developed strategies for optimizing the supply of masks and recommended using homemade cloth masks when no medical masks are available (*1*). However, no guidance is provided for cleaning and decontamination of cloth masks, although standard washing in hot water with soap should be adequate.

## Factors to Consider when Using Cloth Masks to Protect Wearers and to Prevent Spread of Infection during the COVID-19 Pandemic 

The primary transmission routes for severe acute respiratory syndrome coronavirus 2 (SARS-CoV-2) are thought to be inhalation of respiratory droplets and close contact; therefore, WHO recommends wearing medical masks during routine care and using respirators during aerosol-generating procedures and other high-risk situations (*17*). However, SARS-COV-2 is a novel pathogen, and growing evidence indicates the possibility of airborne transmission (*18*–*21*). Recommendations to wear masks to protect the wearer from droplet infections are based on the assumption that droplets travel short distances only, generally 1–2 m. However, of 10 studies of horizontal droplet distance, 8 showed that droplets travel >2 m, in some instances ≈8 m (*22*). A recent study also showed that SARS-CoV-2 may be transmitted up to 4 m (*18*). Therefore, ideally, all frontline healthcare workers should use a respirator. However, demand for personal protective equipment has increased during the COVID-19 pandemic, and respirator shortages in previous pandemics have also been reported (*23*–*26*). If respirators are unavailable, healthcare workers could use a medical mask but may be at increased risk if they do so (*2*). CDC and the European Centre for Disease Prevention and Control initially recommended that all healthcare workers use respirators; however, because of shortages, they later recommended respirator use for high-risk situations only (*27*,*28*). Some countries also recommend sterilizing and decontaminating respirators for reuse; however, limited evidence supports these practices (*29*), and they may not be feasible in low- and middle-income countries. 

During a pandemic, cloth masks may be the only option available; however, they should be used as a last resort when medical masks and respirators are not available (*3*). Cloth mask use should not be mandated for healthcare workers, but some may choose to use them if there are no alternatives (*30*). Protection is affected by proper mask use as well as by selection of fabric and design of the masks for water resistance, filtration, and fit. Current evidence suggests that multilayered masks with water-resistant fabric, high number of threads, and finer weave may be more protective (*3*,*10*). Several studies have examined filtration, but fewer have examined fit or water resistance. Surgical masks are normally rated for fluid resistance, and cloth masks should be too. Masks should be able to prevent a stream of fluid flowing at a pressure of up to 160 mm Hg from seeping through the mask and potentially into the mouth. Furthermore, the degree of fit affects effectiveness because air flows in the direction of least resistance; if gaps are present on the sides of the mask, air will flow through those gaps instead of through the mask.

Cloth masks can be made in large quantities in a short time. They can be reused after being decontaminated by various techniques, ideally washing in hot water with soap. Other methods or products include using bleach, isopropyl alcohol, or hydrogen peroxide; autoclaving or microwaving; and application of ultraviolet radiation or dry heat (*16*). Unlike disposable medical masks and respirators, the material of cloth masks is unlikely to degrade from standard decontamination procedures. However, hospitals will have the extra burden of cleaning and decontaminating used masks. If healthcare workers perform decontamination themselves, they may fail to wash masks frequently enough and may risk self-contamination (*31*). 

The general public can use cloth masks to protect against infection spread in the community. In community settings, masks may be used in 2 ways. First, they may be used by sick persons to prevent spread of infection (source control), and most health organizations (including WHO and CDC) recommend such use. In fact, a recent CDC policy change with regard to community use of cloth masks (*1*) is also based on high risk for transmission from asymptomatic or presymptomatic persons (*32*). According to some studies, ≈25%–50% of persons with COVID-19 have mild cases or are asymptomatic and potentially can transmit infection to others. So in areas of high transmission, mask use as source control may prevent spread of infection from persons with asymptomatic, presymptomatic, or mild infections. If medical masks are prioritized for healthcare workers, the general public can use cloth masks as an alternative. Second, masks may be used by healthy persons to protect them from acquiring respiratory infections; some randomized controlled trials have shown masks to be efficacious in closed community settings, with and without the practice of hand hygiene (*33*). Moreover, in a widespread pandemic, differentiating asymptomatic from healthy persons in the community is very difficult, so at least in high-transmission areas, universal face mask use may be beneficial. The general public should be educated about mask use because cloth masks may give users a false sense of protection because of their limited protection against acquiring infection (*16*). Correctly putting on and taking off cloth masks improves protection (Table). Taking a mask off is a high-risk process (*34*) because pathogens may be present on the outer surface of the mask and may result in self-contamination during removal (*31*). 

**Table:** Recommendations with regard to cloth masks

Activity	Details
Making cloth masks	• Select a fabric with high thread count and fine weave. • If using t-shirt material, cotton blend (*10*) may be better than pure cotton. • Hybrid fabrics such as cotton–silk, cotton–chiffon, or cotton–flannel may be good choices (*10*). • Select a fabric that is water resistant. • Use a minimum of 2–3 layers, preferably with batting between the layers. • Design a mask that fits and seals around the face. • Use ties rather than ear loops because ties provide better fit.
Putting on a cloth mask	• Wash your hands with soap and water or alcohol-based hand sanitizers. • Take a clean and dry cloth mask. • Place and hold the mask over your nose and mouth. Tie upper strings first at the back of your head and then the lower set at the base of your neck. If cloth mask has loops, hold the mask over your nose and mouth and tie ear loops. • If mask has pleats, unfold the mask from top and bottom so it covers your nose, mouth, and chin. • Do not touch the outer layer of face masks during use.
Taking off a cloth mask	• Wash your hands • Do not touch the outer surface of the face mask while removing. • Untie the lower strings first and then upper strings. In case of ear loops, remove ear loops first and then remove the mask. • Place the mask in a plastic zipper-sealed bag until it can be decontaminated. • Wash your hands again after removing the mask.
Caring for masks	• Have at least 2 masks per person, and wash masks with soap and water daily. • Cloth masks can be used for an extended period as long as they are not wet or soiled, but do not reuse them unless washed and cleaned.

## Future Research Directions

More research on cloth masks is needed to inform their use as an alternative to surgical masks/respirators in the event of shortage or high-demand situations. To our knowledge, only 1 randomized controlled trial (*4*) has been conducted to examine the efficacy of cloth masks in healthcare settings, and the results do not favor use of cloth masks. More randomized controlled trials should be conducted in community settings to test the efficacy of cloth masks against respiratory infections. According to the US Institute of Medicine, National Academy of Sciences, more research on the engineering design of cloth masks to enhance their filtration and fit is needed (*16*). Moreover, various methods for decontaminating cloth masks should be tested.

## Conclusions

The filtration, effectiveness, fit, and performance of cloth masks are inferior to those of medical masks and respirators. Cloth mask use should not be mandated for healthcare workers, who should as a priority be provided proper respiratory protection. Cloth masks are a more suitable option for community use when medical masks are unavailable. Protection provided by cloth masks may be improved by selecting appropriate material, increasing the number of mask layers, and using those with a design that provides filtration and fit. Cloth masks should be washed daily and after high-exposure use by using soap and water or other appropriate methods. 
